# The Complex Interplay Between Emotion Regulation and Work Rumination on Exhaustion

**DOI:** 10.3389/fpsyg.2019.01978

**Published:** 2019-08-29

**Authors:** Martin Geisler, Sandra Buratti, Carl Martin Allwood

**Affiliations:** Department of Psychology, University of Gothenburg, Gothenburg, Sweden

**Keywords:** emotion regulation, work rumination, detachment from work, exhaustion, mediation, moderation

## Abstract

This study examined the interplay between emotion regulation strategies (reappraisal and suppression) and work-related rumination (affective work rumination and detachment from work) on exhaustion. In all, 1985 participants from three human service occupations (psychologists, teachers, and ministers) completed the web-based survey. The results showed that reappraisal and detachment from work had a negative relation to exhaustion, whereas the relation between suppression and affective work rumination to exhaustion were positively directed. Moreover, results of mediation analyses showed that the associations between emotion regulation strategies and exhaustion were mediated by work-ruminative tendencies. However, results of moderation analyses did not support that work-ruminative tendencies have a conditional effect (i.e., moderate) on the relationship between emotion regulation strategies and exhaustion. The results suggest that work-ruminative tendencies are best understood as a mediator of the emotion regulation strategies – exhaustion relationship. Thus, the study contribute to the understanding of the strategies (and combination of strategies) people use to reduce exhaustion by adding novel insights into the role of person characteristics in the recovery process. We discuss our results in relation to previous research, provide recommendations for future research, and note possible practical implications.

## Introduction

Work-related stress and exhaustion are serious problems in many societies (Eurofound, 2018). In Sweden, the prevalence of work-related stress and associated long-term sick-leaves have increased in recent years (Swedish Social Insurance Agency, 2016). Moreover, approximately 30% of the Swedish working population (ca. 1, 5 million people) report symptoms of depression and exhaustion due to their work (Swedish Work Environment Authority, 2018). While exhaustion is prevalent in many occupations, human service workers seem to be especially exposed and affected – proposedly due to the pronounced emotional aspects in these types of occupations (e.g., Maslach and Jackson, 1981; Mor Barak et al., 2001; Dollard et al., 2003; Hasenfeld, 2010).

Tendencies to engage in work rumination, preservative thoughts about work and work-related issues outside one’s working hours or to be able to detach from work and refrain from job-related thoughts during non-work time, are important for workers’ health and well-being (e.g., Sonnentag et al., 2010; Cropley et al., 2017; Bennet et al., 2018; van Laethem et al., 2018). Yet, paradoxically, when job stressors are high and recovery is needed the most, recovery processes often seem to be impaired. The “recovery paradox” suggests a complex interplay between job stressors, recovery, and well-being (Sonnentag, 2018). Moreover, though it has been noted that detachment from work do not simply occur but likely requires self-regulatory effort (Zijlstra et al., 2014; Sonnentag and Fritz, 2015), investigations of antecedents or ameliorative factors for detachment from work have primarily been attending to work-characteristics or leisure activities (e.g., Bennet et al., 2018), whereas person characteristics have received a more limited attention (Wendsche and Lohmann-Haislah, 2017).

One characteristic that is relevant to investigate for understanding variations in detachment from work is emotion regulation strategies. Emotion regulation strategies refer to the processes people use in order to shape and manage their emotional experiences and expressions. Previous research reports that emotion regulation strategies relate to mental health and occupational well-being (e.g., Wiltink et al., 2011; Webb et al., 2012; Hu et al., 2014; Ioannidis and Siegling, 2015). Given the pronounced emotional components in human-service work, the role of emotion regulation strategies is of particular importance for work-related rumination and detachment from work among human service workers.

The present study investigated the interplay between emotion regulation strategies (*reappraisal* and *suppression*) and work rumination tendencies (*detachment from work* and *affective work-rumination*), in relation to exhaustion. Specifically, as previous research suggest that work-ruminative tendencies partially mediate the relation between independent variables and exhaustion (e.g., Bennet et al., 2018), whereas existing evidence proposes that work-ruminative tendencies seem to moderate the specific relation between emotion regulation and exhaustion (Blanco-Donoso et al., 2017), we examined if work-ruminative tendencies mediate, or moderate, the relationship between emotion regulation strategies and exhaustion. The aim of this investigation was to contribute to the understanding of how person characteristics are involved in the recovery process (Wendsche and Lohmann-Haislah, 2017; Sonnentag, 2018) and the strategies people use to reduce exhaustion (Demerouti, 2015).

### Affective Work Rumination and Detachment From Work

Work rumination refers to unintentionally initiated, preservative thoughts without obvious external cues that in turn hinders recovery and increase physical symptoms, anxiety, and depression (Cropley and Purvis, 2003). Work rumination can be manifested in different ways. For instance, work rumination can be expressed by affective work rumination, in terms of persevering thoughts about work that elicits negative affect, or in terms of detachment from work, referring to the ability to mentally distancing oneself from work and to refrain from work-related rumination during non-work time (Cropley et al., 2012, 2017). It has been suggested that when people do not detach from work, they tend to be occupied with negative work-related thoughts (Sonnentag, 2018). Thus, detachment from work and affective work rumination can be regarded “… *as opposite ends of one dimension of mental distancing from work during off-job time*” (Wendsche and Lohmann-Haislah, 2017, p. 2).

Research has convincingly demonstrated that when workers confront high levels of job demands and strains at work, they experience greater difficulties to detach from work during non-work time (e.g., Cropley and Purvis, 2003; Sonnentag et al., 2010; Sonnentag and Fritz, 2015). Furthermore, in demanding work settings, people are often required to exert various types of self-control (e.g., desist to act on impulse, inhibit expressions of emotions, or restrict attention away from distractions: Sonnentag et al., 2010). Such demands on self-control can mediate the effect of other types of job demands (e.g., qualitative demands or workload) on workers’ health and well-being (Diestel and Schmidt, 2009). Nonetheless, though it has been put forward that people likely are required to assert some kind of effort in order to disconnect ruminative thoughts and feelings about work during non-work time (Sonnentag and Fritz, 2015) and that recovery from work is a process that seems to be highly contingent on self-regulatory capacity (Zijlstra et al., 2014), basic aspects of self-control have been largely overlooked for the understanding of the recovery process (Sonnentag, 2018).

In a recent meta-analysis, Bennet et al. (2018) noted that previous research has reported that work rumination has a restricted explanatory value for fatigue (i.e., exhaustion) above and beyond the explanation provided by work characteristics. However, the result of the meta-analysis showed that when work-related rumination tendencies were included as a partial mediator in the statistical model the explanation of variance increased substantially (Bennet et al., 2018). Thus, work-related rumination is a distinct factor of specific importance in order to understand occupational health and well-being (Sonnentag and Fritz, 2015; Wendsche and Lohmann-Haislah, 2017; Sonnentag, 2018).

### Emotion Regulation Strategies

People use emotion regulation strategies in order to shape and manage their emotional experiences and expressions. Emotion regulation is distinguished from mere coping, as emotion regulation may be deployed to increase or decrease both negative and positive emotions whereas coping focuses on decreasing negative affect (Gross, 1998, 2001, 2015). Much research on emotion regulation strategies has been based on the process model of emotion regulation (Gross, 1998, 2015). In brief, the process model of emotion regulation proposes that the impact or consequences of various emotion regulation strategies differs as an effect of when they are deployed in the emotion-generation process (Gross, 2015).

Even though many different emotion regulation strategies are distinguished (e.g., situation selection, distraction, situation modification), research has mainly attended to the differentiation and effect of two specific strategies: suppression and reappraisal. Suppression (cf. response modulation) refers to the behavioral down-regulation of experienced emotions (positive or negative), whereas reappraisal is a cognitive process whereby a person tries to alter (increase or decrease) an emotional response by revising or reevaluate how a situation is appraised (Gross, 1998, 2015). Overall, research has reported that reappraisal seems to be an efficient and advantageous strategy, whereas suppression is a more maladaptive strategy that often is related to adverse outcomes (e.g., Gross and John, 2003; Wiltink et al., 2011; Ioannidis and Siegling, 2015; Chevronsky and Hunt, 2017).

However, this simplified differentiation and understanding of the effects of reappraisal and suppression needs to be more nuanced. For instance, suppression may be an effective regulation strategy with regard to the expression of emotions, but a maladaptive regulation strategy with respect to the experience of emotions and emotion-eliciting events (e.g., Webb et al., 2012). Furthermore, people seem to select different emotion-regulation strategies depending on the situation, the context, or how intense an emotion is experienced (Sheppes et al., 2012). Thus, it has been suggested that the primary focus should not be on differences in the effectiveness of specific emotion regulation strategies, but on individual differences in the regulatory flexibility in terms of sensitivity to context, availability of a wider repertoire of strategies, and responsiveness to feedback (Bunnano and Burton, 2013). In addition, it is important to note that, in their everyday, people do not simply use a single emotion regulation strategy but often deploy a combination of different strategies simultaneously (Gross, 2015).

Emotion regulation strategies are processes that interact with social factors to assert an influence on mental health. For instance, the nature of close relationships influence the emotion regulation strategies people use and, in turn, explain if emotion regulation strategies relate to mental health impairments like depressive symptoms (Marroquín and Nolen-Hoeksema, 2015). Moreover, the relation between emotion regulation and strain can depend on interpersonal processes and social interactions, and may be understood by attending to the type of emotion regulation deployed, the type of emotion regulated, and the type of response received in social interactions (Côte, 2005, see also Chevronsky and Hunt, 2017). The social associations and dependencies of emotion regulation strategies illustrate their importance in relation to how people experience and relate to work – especially in human service professions (e.g., Guy et al., 2010; Buruck et al., 2016).

### Emotion Regulation and Emotion Regulation Strategies in Relation to Work

Explorations focusing on the effect of emotions and the regulation of emotions at work was primarily initiated within research on emotional labor. In brief, research on emotional labor differentiates between emotion regulation requirements in terms of “surface acting” (i.e., regulating emotional expressions) and “deep acting” (i.e., consciously modifying ones’ feelings in order to express desired emotions), and how these requirements effect workers’ health and well-being (Grandey, 2000). The emotional labor perspective on emotion regulation at work is of relevance for customer interactions in the service industry (e.g., Judge et al., 2009; Hülsheger et al., 2015). Still, the emotional labor perspective focuses on interpersonal emotion regulation (i.e., the regulation of emotional experiences in line with professional/organizational requirements: e.g., Côte, 2005) whereas the process model definition of emotion regulation focuses on intrapersonal emotion regulation (i.e., the regulation of emotional experience and expression in relation and correspondence to personal goals: see Gross, 2015; English et al., 2017; Troth et al., 2017; however, cf. Grandey and Melloy, 2017). Thus, with respect to how emotion regulation strategies influence in what way people try to manage their emotional experiences and exhaustion in relation to work, and how well they are able to detach from work during non-work time, the process model definition of emotion regulation (i.e., intrapersonal processes) can be considered more suitable as compared to the emotional labor perspective.

The importance of emotion regulation and emotion regulation strategies has been investigated within the context of human service. For instance, in a sample of clinical psychologists, general emotion regulation difficulties (i.e., problems to understand, recognize, and regulate emotions) were found to be associated with higher levels of self-reported stress (Finlay-Jones et al., 2015). Moreover, in a sample of the clergy, training how to regulate and cope with the emotional aspects of work (i.e., counseling training) reduced the negative effect of emotional labor on psychological distress (Kinman et al., 2011). The importance of the emotion regulation strategies reappraisal and suppression has also been reported in relation to teachers’ occupational health and well-being. Taxer and Gross (2018) reported that teachers were most inclined to down regulate negative emotions through the use of suppression. Furthermore, Jiang et al. (2016) found that teachers’ self-reflections of emotion regulation strategies (interviews) corresponded with students’ perceptions (questionnaire); teachers’ use of suppression was related to less desirable student evaluations (e.g., annoyed, distracted), and teachers’ use of reappraisal was related to more desirable (e.g., inspired, tender) student evaluations. In addition, Yin et al. (2018) reported that teachers’ use of suppression was positively related to teachers’ levels of anxiety and depression, whereas reported use of reappraisal was positively related to teachers’ enthusiasm and contentment.

### Emotion Regulation Strategies and Work Rumination in Relation to Exhaustion

Based on the existent evidence, emotion regulation strategies and work-ruminative tendencies are important for understanding fatigue and exhaustion. Though emotion regulation and detachment are distinct constructs, they may be intertwined and assert an effect on exhaustion through a complex interplay. Indeed, Gross and John (2003) investigated the relation between emotion regulation strategies (i.e., reappraisal and suppression) and rumination (mood and self-centered) in the development of the Emotion Regulation Questionnaire (ERQ: Gross and John, 2003), and reported that suppression was related to more rumination and reappraisal to less rumination.

Yet, people use different strategies to minimize exhaustion and its unfavorable effects (Demerouti, 2015; Sonnentag, 2018), and it is unclear if, or how, work-ruminative tendencies are involved in the emotion regulation strategies – exhaustion relationship. On the one hand, work rumination is generally considered as a *mediator* of the relationship between work characteristics and health or well-being (e.g., Bennet et al., 2018). One the other hand, to the best of our knowledge, only one previous study has investigated the interplay between emotion regulation and work rumination on exhaustion. In a human service sample (nurses), Blanco-Donoso et al. (2017) found that detachment from work *moderated* the effect of emotion regulation difficulties on exhaustion. Still, in the discussion of their study results, Blanco-Donoso and colleagues encouraged future research to investigate plausible mediation models for the interplay between emotion regulation and recovery on exhaustion. Thus, the present understanding of the interplay between emotion regulation and work rumination on exhaustion is unclear and more research is needed.

### The Present Study

With regard to emotion regulation strategies, reappraisal seems to be related with lower levels, and suppression with higher levels, of exhaustion (Jiang et al., 2016; Yin et al., 2018). Furthermore, to be able to detach from work, and to disengage ruminative thoughts and feelings about work during non-work time, is important for an efficient recovery process and related to lower levels of exhaustion (Sonnentag et al., 2010; Bennet et al., 2018). Based on these considerations, we propose:

*Hypothesis 1.* Reappraisal will have a positive association to (a) detachment from work, but a negative association to (b) affective work rumination and (c) exhaustion.
*Hypothesis 2.* Suppression will have a negative association to (a) detachment from work, but a positive association to (b) affective work rumination and (c) exhaustion.
*Hypothesis 3.* Detachment from work will have a negative association to exhaustion.
*Hypothesis 4.* Affective work rumination will have a positive association to exhaustion.

Moreover, the present understanding is rather deficient with regard to the interplay between the various types of strategies that people use to reduce exhaustion and improve recovery (Demerouti, 2015; Sonnentag, 2018). Thus, it is not clear if certain combinations of strategies that people utilize to minimize exhaustion are more effective in comparison to the use of a single strategy (Demerouti, 2015). In addition, only limited attention has been given to the role of person characteristics in the recovery process (Wendsche and Lohmann-Haislah, 2017). The aim of the present study is to contribute by investigating the indirect effects of emotion regulation strategies on exhaustion through work-ruminative tendencies (i.e., mediation), as well as the conditional effect of work-ruminative tendencies on the association between emotion regulation strategies on exhaustion (i.e., moderation). Based on the suggestions and results reported in previous research (Blanco-Donoso et al., 2017; Bennet et al., 2018) we propose:

*Hypothesis 5.* Work-ruminative tendencies (i.e., detachment from work; affective work rumination) will mediate the association between emotion regulation strategies (i.e., reappraisal; suppression) and exhaustion.
*Hypothesis 6.* Work-ruminative tendencies (i.e., detachment from work; affective work rumination) will moderate the association between emotion regulation strategies (i.e.,reappraisal; suppression) and exhaustion.

## Materials and Methods

### Participants and Procedure

The data for the present study was collected as part of the second-wave of a longitudinal (three-wave) study. In the first-wave, a total of 13,513 human service workers from all over Sweden were invited and 2,835 participated (*response rate* = 21%). For the second wave, invitations were sent out to participants with a minimum 75% completion rate in the first wave (2,638) minus those participants (*n* = 11) who had declined from further participation (e.g., due to retirement, parental leave). Out of 2,627 invited human service workers in the second wave, 1,985 participated (*response rate* = 76%; *mean age* = 47 years; *SD age* = 10.6 years; *women* = 73%). Information regarding emotion regulation strategies were only collected in the second wave. The data was collected by use of a web-based survey and answered by participants from three human-service occupations in Sweden: *ministers* (33%), *psychologists* (31%), and *teachers* (36%). Invitations to participate in the study were sent out by email which provided information about the purpose of the study, confidentiality, and contact information to the researchers. Each email contained an individual link to the web-based survey. The survey was open for participation for a period of 3 weeks. Two reminders were sent out by email (one per week), to participants who at that time had not yet completed the survey. The survey took approximately 20–25 min to complete. Participants gave their informed consent by activating the individual link. The study was approved by the Regional Ethical review-board, Gothenburg secretariat (dnr: 608-17).

### Measures

#### Reappraisal and Suppression

Emotion regulation strategies were assessed by use of the validated Swedish version (Enebrink et al., 2013) of the ERQ (Gross and John, 2003). The ten items on the ERQ assess reappraisal (6 items) and suppression (4 items) by asking participants to rate the extent to which they typically try to think or behave to change their emotions. Participants provide their answers on a 7-point Likert scale, ranging from “Strongly disagree” (1) to “Strongly agree” (7). Item examples are: “*When I want to feel less negative emotion, I change the way I’m thinking about the situation*” (Reappraisal, α = 0.87); “*I control my emotions by not expressing them*” (Suppression, α = 0.74).

#### Detachment From Work and Affective Work Rumination

Detachment from work and affective rumination were assessed by use of the two respective sub-scales on the Work Rumination Scale (WRS: Cropley et al., 2012). Each subscale is measured by 5 items, which participants respond to on a 5-point Likert scale ranging from “*Very seldom*/*never*” (1) to “*Very often*/*always*” (5). Scores are calculated by the mean score on the respective subscale. Item examples are: “*Do you feel unable to switch off from work?*” (Detachment from work, α = 0.91), and “*Do you become tense when you think about work-related issues during your free time?*” (Affective work rumination, α = 0.92).

#### Exhaustion

Exhaustion was assessed by use of the validated Swedish version (Lundgren-Nilsson et al., 2012) of the Shirom Melamed Burnout Questionnaire (SMBQ: Melamed et al., 1992). The 22 items on the SMBQ measure self-reported exhaustion in terms of physical exhaustion, emotional exhaustion (c.f. listlessness), tension, and cognitive weariness. Each item is rated on a 7-point scale ranging from “*Almost never*” (1) to “*Almost always*” (7). In the present study, the total exhaustion score was used, calculated by the average mean, where higher scores indicate more exhaustion (α = 0.96). Item examples are: “*I feel I am not thinking clearly*” and “*I feel alert*” (reverse scored).

#### Data Analyses

Data were analyzed by use of correlation analyses, and mediation regression analyses using SPSS (vers. 25) and the PROCESS macro (vers. 2.16.3), as well as moderation analyses using the PROCESS macro (vers. 3.2). *Mediation models 1–2* tested the indirect effects of reappraisal on exhaustion – through detachment from work and affective work rumination, respectively. *Mediation models 3–4* tested the indirect effects of suppression on exhaustion – through detachment from work and affective rumination, respectively. Furthermore, *Moderation models 1–2* tested the moderating effect of detachment from work, and *Moderation models 3–4* the moderating effect of affective work-rumination, for the respective relationship between reappraisal and suppression on exhaustion. The mediation analyses used a 95% confidence interval (CI) with 5000 bootstrap, bias corrected (BCa) (Zhao et al., 2010). Furthermore, the effect size for each mediation model was calculated by the proportion mediated (*P*_*M*_) which is considered an appropriate effect-size measure for mediation when the sample size is above 500 and the independent variable is continuous (Preacher and Kelley, 2011; Miocevic et al., 2018). In the moderation analyses, both the independent and the moderating variables were mean centered and simple slopes were calculated based on sample values (i.e., estimates of population values: *M* – 1 *SD*, and *M* + 1 *SD*), using a 95% CI with 5000 bootstrap, bias corrected (BCa) (Hayes, 2018).

## Results

### Descriptive Statistics and Correlations

Descriptive statistics and correlations between all variables are reported in Table 1. Overall, the present sample reported moderate levels of detachment from work/affective work rumination. Furthermore, the results indicated that the participants report to regulate their emotions by more frequent use of reappraisal, as compared to suppression. Finally, the mean level of exhaustion was rather high (3.5) compared to the mean value (2.9) reported in a sample of the Swedish working population (Norlund et al., 2010). Negative correlations were found between detachment from work and reappraisal to exhaustion, whereas the correlations between affective work rumination and suppression to exhaustion were positive. Stronger correlations to exhaustion were observed for detachment from work and affective rumination, as compared to the two emotion regulation strategies reappraisal and suppression.

**TABLE 1:** Descriptive statistics and correlations.

	***M***	***SD***	**Observed range**	**Skewness**	**1**	**2**	**3**	**4**	**5**
1. Affective work rumination	2.76	0.92	1–5	0.16	–				
2. Detachment from work	3.12	0.91	1–5	−0.17	−0.697^∗^	–			
3. Reappraisal	4.33	6.42	1–7	−0.49	−0.162^∗^	0.166^∗^	–		
4. Suppression	2.87	4.37	1–7	0.46	0.140^∗^	−0.165^∗^	0.026	–	
5. Exhaustion	3.50	1.21	1–7	0.30	0.682^∗^	−0.559^∗^	−0.220^∗^	0.172^∗^	–

*^∗^*p* < 0.001.*

### Mediation Analyses

#### Work Ruminative Tendencies as Mediators of the Relationship Between Emotion Regulation Strategies and Exhaustion

**FIGURE 1:**
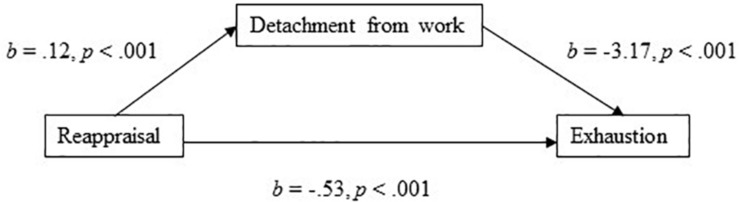
Detachment from work as a mediator of the association between reappraisal and exhaustion.

The model with reappraisal as the independent variable and detachment from work as the mediator, explained 32% of the variance in exhaustion. Hypothesis 1 proposed that reappraisal will have a positive association to (a) detachment from work, but a negative association to (c) exhaustion. Furthermore, Hypothesis 5 proposed that work-ruminative tendencies would partially mediate the association between emotion regulation strategies and exhaustion. In support of Hypothesis 1(c), results show that reappraisal had a significant negative direct effect on exhaustion (*b* = −0.53, 95% CI [−0.69, −0.37], *t* = −6.44, *p* < 0.001). In the mediation model (Figure 1), the total effect of reappraisal on exhaustion was clear and negatively directed (*b* = −0.901, 95% CI [−1.0889, −0.714], *t* = −9.45, *p* < 0.001), and reappraisal had a positive association (*b* = 0.12, *p* < 0.001) to detachment from work (Hypothesis 1a). In addition, and in support of Hypothesis 5, the indirect effect of reappraisal on exhaustion through detachment from work was significant (*b* = −0.376, 95% BCa CI [−0.487, −0.264]). The effect-size for the mediation model, in terms of the proportion mediated, was *P*_*M*_ = 0.417 – meaning that 42% of the effect of reappraisal on exhaustion was mediated by detachment to work.

**FIGURE 2:**
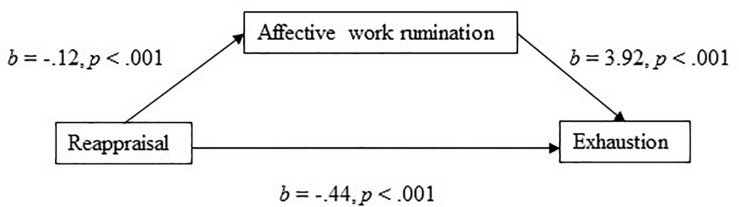
Affective work rumination as a mediator of the association between reappraisal and exhaustion.

The model with reappraisal as the independent variable and affective work rumination as the mediator explained 48% of the variance in exhaustion. Again, reappraisal had a significant negative direct effect on exhaustion (*b* = −0.44, 95% CI [−0.58, −0.30], *t* = −6.08, *p* < 0.001). In the mediation model (Figure 2), the total effect of reappraisal on exhaustion was clear and negatively directed (*b* = −0.902, 95% CI [−1.090, −0.715], *t* = −9.45, *p* < 0.001), and there was a significant indirect effect (Hypothesis 5) of reappraisal on exhaustion through affective work rumination (*b* = −0.467, 95% BCa CI [−0.603, −0.333]). The effect-size for the mediation model was *P*_*M*_ = 0.518, that is, 52% of the effect of reappraisal on exhaustion was mediated by affective work rumination. Supporting Hypothesis 1(b), reappraisal had a negative association (*b* = −0.12, *p* < 0.001) to affective work rumination.

**FIGURE 3:**
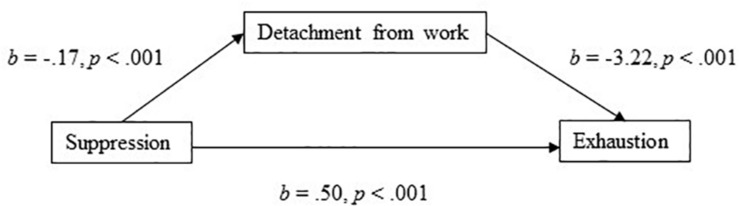
Detachment from work as a mediator of the association between suppression and exhaustion.

The model with suppression as the independent variable and detachment from work as the mediator explained 31% of the variance in exhaustion. In support of Hypothesis 2(c), suppression had a positive direct effect on exhaustion (*b* = 0.50, 95% CI [0.27, 0.74], *t* = 4.16, *p* < 0.001). In the mediation model (Figure 3), the total effect of suppression on exhaustion was clear and positively directed (*b* = 1.048, 95% CI [0.771, 1.326], *t* = 7.41, *p* < 0.001), and the results showed that there was a significant indirect effect (Hypothesis 5) of suppression on exhaustion through detachment from work (*b* = 0.546, 95% BCa CI [0.378, 0.707]). For this model, the effect-size was *P*_*M*_ = 0.521, thus, 52% of the effect of suppression on exhaustion was mediated by detachment from work. In addition (Hypothesis 2c), the association between suppression and detachment from work was negative (*b* = −0.17, *p* < 0.001).

**FIGURE 4:**
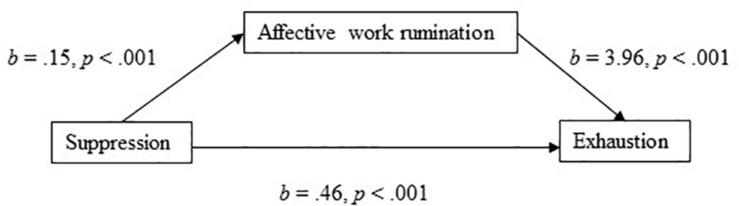
Affective work rumination as a mediator of the association between suppression and exhaustion.

Furthermore, the model with suppression as the independent variable and affective work rumination as the mediator explained 47% of the variance in exhaustion. Suppression had a significant positive direct effect on exhaustion (*b* = 0.46, 95% CI [0.25, 0.67], *t* = 4.35, *p* < 0.001). In the mediation model (Figure 4), the total effect of suppression on exhaustion was clear (*b* = 1.053, 95% CI [0.755, 1.331], *t* = 7.43, *p* < 0.001). The results showed that there was a significant indirect effect (Hypothesis 5) of suppression on exhaustion through affective work rumination (*b* = 0.594, 95% BCa CI [0.403, 0.806]). The effect-size was *P*_*M*_ = 0.564, meaning that 56% of the effect of suppression on exhaustion was mediated by affective work rumination. Also, in support of Hypothesis 2b, suppression had a positive association (*b* = 0.15, *p* < 0.001) to affective work rumination.

### Moderation Analyses

#### Work Ruminative Tendencies as Moderators of the Relationship Between Emotion Regulation Strategies and Exhaustion

**FIGURE 5:**
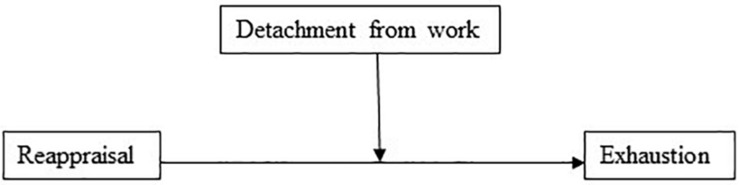
Hypothesized model: detachment from work as a moderator of the reappraisal to exhaustion relationship.

**FIGURE 6:**
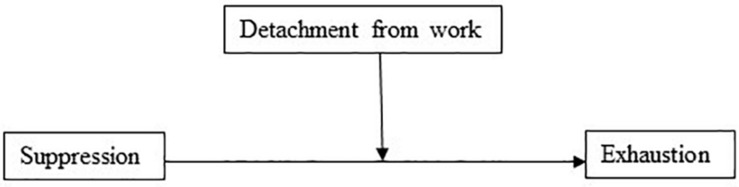
Hypothesized model: detachment from work as a moderator of the suppression to exhaustion relationship.

**FIGURE 7:**
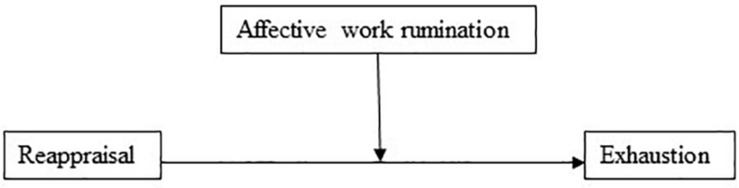
Hypothesized model: affective work rumination as a moderator of the reappraisal to exhaustion relationship.

**FIGURE 8:**
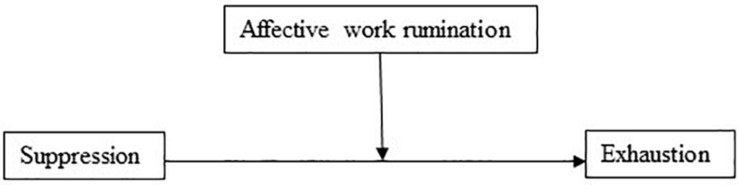
Hypothesized model: affective work rumination as a moderator of the suppression to exhaustion relationship.

Hypothesis 6 proposed that work-ruminative tendencies (i.e., detachment from work; affective work rumination) will moderate the association between emotion regulation strategies (i.e., reappraisal; suppression) and exhaustion. The tested moderation models are illustrated in Figures 5–8. The results (Table 2) showed that emotion regulation strategies had a significant relationship to exhaustion. Reappraisal was related to lower levels of exhaustion, whereas suppression was related to higher levels of exhaustion. Furthermore, detachment from work was related to lower levels of exhaustion (model 1–2), and affective work rumination was related to higher levels of exhaustion (model 3–4). However, no support was provided for Hypothesis 6, as none of the tested interactions (Figures 5–8), positing a moderating effect of work-ruminative tendencies for the relationship between emotion regulation strategies and exhaustion, were found to be significant (Table 2).

**TABLE 2:** Moderation analyses testing the conditional effect of detachment from work and affective work rumination on the relationship between reappraisal and suppression to exhaustion.

**Exhaustion**									
	
	***R***	**Δ*R***^2^	***F***	**Coeff**	**SE**	***t***	***p***	**LLCI**	**ULCI**
**Model 1**	0.573	0.328	(3, 1897) 308.604^∗^						
Constant				3.502	0.023	151.402	0.000	3.457	3.547
Reappraisal (R)				−0.147	0.022	–6.734	0.000	−0.189	−0.104
Detachment from work (DW)				−0.712	0.025	–28.029	0.000	−0.762	−0.663
Interaction (R × DW)				0.005	0.022	0.231	0.817	−0.037	0.047
**Model 2**	0.565	0.319	(3, 1898) 295.926^∗^						
Constant				3.501	0.023	150.384	0.000	3.455	3.546
Suppression (S)				0.090	0.022	4.148	0.000	0.047	0.132
Detachment from work (DW)				−0.724	0.026	–28.366	0.000	−0.774	−0.674
Interaction (S × DW)				−0.009	0.021	–0.421	0.674	−0.051	0.033
**Model 3**	0.691	0.478	(3, 1897) 577.966^∗^						
Constant				3.498	0.020	171.540	0.000	3.458	3.538
Reappraisal (R)				−0.129	0.019	–6.571	0.000	−0.163	−0.088
Affective work rumination (AR)				0.872	0.022	39.456	0.000	0.829	0.915
Interaction (R × AR)				−0.030	0.019	–1.560	0.119	−0.067	0.008
**Model 4**	0.687	0.472	(3, 1898) 565.243^∗^						
Constant				3.497	0.020	171.147	0.000	3.457	3.537
Suppression (S)				0.082	0.019	4.354	0.000	0.045	0.119
Affective work rumination (AR)				0.882	0.022	39.852	0.000	0.839	0.926
Interaction (S × AR)				0.034	0.019	1.782	0.075	−0.003	0.070

*^∗^*p* < 0.001.*

## Discussion

Work-related stress and exhaustion is a serious problem (Eurofound, 2018), that is especially evident among human service workers (Maslach and Jackson, 1981; Mor Barak et al., 2001; Dollard et al., 2003; Hasenfeld, 2010). To detach from work, and to disconnect ruminative thoughts and feelings about work during non-work time, is important in order to reduce levels of exhaustion (Sonnentag and Fritz, 2015; Bennet et al., 2018). However, though people use different strategies to minimize the impact of work in order to prevent exhaustion (Demerouti, 2015), and detachment from work likely requires efforts of self-control (Sonnentag, 2018), the role of person characteristics has received limited attention in previous research on the recovery process (Wendsche and Lohmann-Haislah, 2017).

In the present study, we considered the role of person characteristics in the recovery process by attending to emotion regulation strategies, and investigated the interplay between emotion regulation strategies and work-ruminative tendencies in relation to exhaustion. Specifically, by mediation analyses, we investigated the indirect effects between emotion regulation strategies (*reappraisal* and *suppression*) and exhaustion – through work-ruminative tendencies (detachment from work and affective work rumination). Furthermore, by moderation analyses, we tested the conditional effect of work-ruminative tendencies for the emotion regulation strategies on exhaustion relationships. In all, our Hypotheses 1–4 were supported, proposing expected directions in associations between the independent variables and in their respective association to the dependent variable. Furthermore, the results provided support for Hypothesis 5, proposing that work-ruminative tendencies will mediate the association between emotion regulation strategies and exhaustion. However, the results did not provide support for Hypothesis 6, positing that work-ruminative tendencies will moderate the association between emotion regulation strategies and exhaustion.

Thus, the results showed that work-ruminative tendencies mediate the association between emotion regulation strategies and exhaustion. Accordingly, the results support the notion that the relationship between emotion regulation strategies and exhaustion is better understood if the mediating effect of work-ruminative tendencies is considered and included. Furthermore, the results did not provide support for the idea that work-ruminative tendencies moderate the relationship between emotion regulation strategies and exhaustion. Hence, our results do not support previous research, reporting that work-ruminative tendencies (cf. detachment and relaxation) moderate the effect of emotion regulation strategies (cf. difficulties in emotion regulation) on exhaustion (Blanco-Donoso et al., 2017). Instead, our results are in line with the general assumption and results reported by previous research, in that work-ruminative tendencies seem to be best understood as a mediator for the relationship between various characteristics (i.e., work/person characteristics) to outcomes in terms of health and well-being (e.g., Bennet et al., 2018).

In sum, the results of our study demonstrate a rather complex interplay between emotion regulation strategies and work-ruminative tendencies in relation to exhaustion. The results show that the use of reappraisal is associated with lower levels of exhaustion, as well as with more detachment from work and less affective work rumination. In contrast, the results show that the use of suppression is related to higher levels of exhaustion, as well as to less detachment from work but more affective work rumination. Moreover, our results contribute by demonstrating that the associations between reappraisal and suppression on exhaustion is in part explained by variations in work-ruminative tendencies. That is, an inclination to regulate emotions by changing the way one thinks about the situation (i.e., the situation in relation to personal goals and the emotions evoked by discrepancies between the situation and the goal; Gross, 2015) is associated with lower levels of exhaustion, and the association is in part explained by (more) detachment from work and (less) affective work rumination. In some contrast, a suppressive disposition (i.e., trying to control and inhibit the expression of experienced emotions) is related with higher levels of exhaustion, and this association is partially explained by (less) detachment from work and (more) affective work rumination.

In general, our results are in line with the reports from previous research, by adding support to the idea that a disposition to manage emotions and emotional reactions in a constructive manner seem to enhance recovery (Smit and Barber, 2016). As reappraisal was found to have favorable associations whereas suppression did not, our results do not support the suggestion that the expected effects of reappraisal and suppression needs to be more nuanced (e.g., Webb et al., 2012; Bunnano and Burton, 2013). Still, it is important to consider that the focus of the present study is on peoples’ thoughts and emotions about work during non-work time, not at work (cf. Miller et al., 2007), or in their life in general (e.g., Gross and John, 2003; Wiltink et al., 2011; Ioannidis and Siegling, 2015).

Furthermore, our study contributes by demonstrating the role of emotion regulation strategies as a person characteristic of importance for understanding the relationship between work-related rumination and exhaustion (Wendsche and Lohmann-Haislah, 2017). However, more research using more comprehensive models (i.e., including additional variables) and study designs (e.g., longitudinal methods) is needed in order to draw conclusions about the effectiveness of various emotion regulation strategies or to make causal inferences. Of note, another plausible model is that the nature of the interrelationship/interplay between emotion regulation strategies and work-ruminative tendencies may be cyclic or reciprocal (cf. gain and loss spirals: Bakker and Demerouti, 2018). Indeed, long-term outcomes of lack of detachment are probably contingent on other factors such as person characteristics (Sonnentag, 2018).

Human service workers confront many different stressors at work, and the work often involves emotional demands and strain which generally have a negative impact on workers’ health and well-being (Mor Barak et al., 2001; Dollard et al., 2003; Hasenfeld, 2010), but may also be positively related to motivation (e.g., Taris and Schreurs, 2009; Bakker and Sanz-Vergel, 2013; Geisler et al., 2019). Notwithstanding, the results of the present study suggests that emotion regulation strategies are important for human service workers’ recovery processes. Relating the result of the present study to previous research on the role of emotion regulation among human service workers, the results support that overtly use of problematic emotion regulation strategies (cf. suppression) is associated with higher levels of stress and exhaustion (Finlay-Jones et al., 2015; Yin et al., 2018). Furthermore, the results suggest that efforts aiming to reduce psychological distress among human service workers by providing training in how to manage emotional aspects of work (Kinman et al., 2011), may ultimately have a beneficial effect on workers’ health through improved recovery processes. That is, training of emotion regulation may foster tolerance of, and flexibility to manage negative thoughts and feelings about work (i.e., reduce affective work rumination) and/or initiate a positive change for how emotional aspects of work are appraised (Buruck et al., 2016).

The results of our study adds insights to the findings reported by Blanco-Donoso et al. (2017) who reported that difficulties in emotion regulation had a direct negative effect on exhaustion, but that psychological detachment and relaxation (cf. work-ruminative tendencies) reduce the negative effect of emotion regulation difficulties on exhaustion. That is, our results do not support that work-ruminative tendencies, neither in terms of detachment from work nor in terms of affective work rumination, moderate the relationship between emotion regulation strategies and exhaustion. Still, the results of our study cannot be directly compared to the results of the study by Blanco-Donoso et al. For instance, aspects of emotion regulation (i.e., difficulties as compared to strategies) or work-ruminative tendencies (psychological detachment and relaxation as compared to detachment from work and affective work rumination) were defined and assessed differently. However, in this regard, it is worth to note that the present study attended to emotion regulation strategies based on the process model of emotion regulation (Gross, 2001, 2015), which is in correspondence with much previous research on emotion regulation in relation to (human service) work (Jiang et al., 2016; Taxer and Gross, 2018; Yin et al., 2018). Furthermore, contrasting the present study to that of Blanco-Donoso et al. (2017), it can be argued that the present results may be more plausible with regard to the possibility of making general inference and expectations to other type of professions. This since the results of the present study are based on a rather large sample (*N* = 1,985; as compared to *N* = 74 in the study by Blanco-Donoso et al.), and as it seems reasonable to expect that constructive regulation of emotion is associated with less exhaustion – through higher detachment from work/less affective work rumination, compared to the expectation that difficulties in emotion regulation would initiate psychological detachment or relaxation. In support of this notion, Sonnentag (2018) noted that “…persons high in neuroticism and high trait negative affectivity react more strongly to job stressors with negative activation that makes it particularly difficult for them to detach from work…” whereas “…persons low on neuroticism (i.e., persons high on emotional stability), are less likely to be affected by job stressors, making it easier for them to initiate and maintain processes that enhance recovery” (p. 177). Thus, our study adds novel insights into the enquiry of the strategies, and combination of strategies, that people use, in order to detach from work (Sonnentag, 2018) and in order to prevent the negative impact of work stressors on exhaustion (Demerouti, 2015).

### Implications

The present study contributes by adding novel insights for how person characteristics in terms of emotion regulation strategies are relevant to consider for understanding the recovery process. Thus, the results show that emotion regulation strategies are important to study in order to understand how people respond to, and try to manage, the impact of work stressors in order to minimize exhaustion (Demerouti, 2015). In terms of practical implications, the results of the present study add insights that could be integrated to the incipient understanding of the benefits associated with psycho-educative efforts to improve workers’ health and well-being through emotion regulation training. The results of the present study indicate that efforts directed to enhance workers’ well-being distress by training workers to manage the emotional aspects of work (e.g., Kinman et al., 2011; Buruck et al., 2016), may ultimately have a beneficial spill-over effects in terms of improved recovery processes. Thus, enhancing emotion regulation strategies could be one way to initiate processes that foster recovery and counteract the negative effects of job stressors (Sonnentag, 2018).

### Limitations and Directions for Future Research

The present study has some limitations. The study is cross-sectional, and based on self-reports, and the results need to be interpreted with the risk of common method bias in mind (Podsakoff et al., 2003). In addition, and importantly, the terminology used to describe mediation analysis (i.e., effects) does not prove or imply causal effects. Hence, if possible, future research should try to replicate and extend the results of the present study by the use of longitudinal design and other measures than self-reports. For instance, high state negative affect has been found to be a factor that undermine recovery (Sonnentag, 2018). Thus, future research could investigate if and how affective states affect the interplay between emotion regulation strategies and work-ruminative tendencies in relation to exhaustion. In addition, future research may also investigate possible gender differences as women tend to report higher levels of rumination compared to men (e.g., Nolen-Hoeksema et al., 1999; Jose and Brown, 2008) but also tend to report to use suppression to a lesser extent than men (e.g., Gross and John, 2003).

Moreover, a longitudinal design could contribute to the understanding of both the short-term and the long-term interplay between emotion regulation strategies and work-ruminative tendencies on exhaustion. For example, the nature of the interrelationship/interplay between emotion regulation strategies and work-ruminative tendencies may be cyclic or reciprocal (cf. gain and loss cycles: Bakker and Demerouti, 2018; see also Blanco-Donoso et al., 2017). Furthermore, the sample in the present study consisted of participants from three different human service professions, and the data was collected at the second wave of a three-wave longitudinal study which may be considered a limitation in terms of self-selection bias (Keiding and Louis, 2016). Still, human service professions share many basic aspects of the job (e.g., Hasenfeld, 2010), and the focus of the present study is on basic (i.e., emotion regulation strategies) and general (detachment from work/affective work rumination) psychological processes which can be regarded to support the possibility to generalize the present findings to other professional groups. Furthermore, based on the available information (Statistics Sweden, 2018), the present sample was rather similar with regard to gender (73% women) and age (mean age = 47 years) compared to the basic population (psychologists: 73% women, mean age = 42 years; teachers: 75% women, mean age = 42 years; ministers: 51% women, mean age = 50 years). Future research should try to replicate the results in other occupational groups and in different cultural settings. Finally, future research could expand the insights of the present study by broadening the scope to include other factors in order to gain a more complete model and understanding of the recovery process (see Sonnentag, 2018).

## Data Availability

The datasets generated for this study are available on request to the corresponding author.

## Ethics Statement

Human Subject Research: The studies involving human participants were reviewed and approved by the Regional Ethical review-board, Gothenburg secretariat. The participants provided their written informed consent to participate in this study.

## Author Contributions

MG, SB, and CA planned and conducted the data collection. MG developed the hypotheses, analyzed the data, and wrote the first draft of the manuscript. SB contributed to the data analyses. SB and CA contributed to the development of the hypotheses, the interpretations, and the discussion of the findings. All authors listed have made direct and intellectual contribution to the article and approved the final version for publication.

## Conflict of Interest Statement

The authors declare that the research was conducted in the absence of any commercial or financial relationships that could be construed as a potential conflict of interest.
